# A130 ENDOSCOPIC ULTRASOUND GUIDED THROMBIN INJECTION: A NOVEL TECHNIQUE IN THE MANAGEMENT OF RECURRENT UPPER GASTROINTESTINAL BLEEDING CAUSED BY GASTRODUODENAL ARTERY PSEUDOANEURYSMS

**DOI:** 10.1093/jcag/gwac036.130

**Published:** 2023-03-07

**Authors:** K Chin Koon Siw, A Kundra, A Chatterjee

**Affiliations:** Division of Gastroenterology, The Ottawa Hospital, Ottawa, Canada

## Abstract

**Background:**

Gastroduodenal artery (GDA) aneurysm is a rare vascular condition that is potentially life-threatening. It is associated with significant morbidity and mortality and most often encountered in patients with chronic pancreatitis. Other etiologies including atherosclerosis, peptic ulcer disease, alcohol abuse, cholecystectomy and absence of celiac axis, have been reported. False and true GDA aneurysms account for about 1.5% of all visceral artery aneurysms however are associated with a risk of rupturing and hemorrhage up to 75%. Mainstay of GDA aneurysm management involves aneurysm repair with open surgery with vessel ligation or endovascular repair with coil embolization or stent placement, depending on the patient’s comorbidities and overall hemodynamic stability. The use of percutaneous thrombin injection via computed tomography (CT) and ultrasound has also been described in the literature and is being increasingly used to promote clot formation and pseudoaneurysm occlusion as a less invasive option.

**Purpose:**

To present a case of endoscopic ultrasound guided thrombin injection as an effective and safe management strategy for GDA pseudoaneurysms.

**Method:**

At this time, there are limited data in the literature on endoscopic ultrasound guided thrombin injection for pseudoaneurysms. To our knowledge, this is the first case report describing a patient with recurrent gastrointestinal bleeding from a duodenal ulcer, who had undergone multiple endoscopic and angiographic procedures, with subsequent development of a gastroduodenal pseudoaneurysm unrelated to pancreatitis which was successfully managed by occluding the pseudoaneurysm with thrombin injection under endoscopic ultrasound guidance. EUS guided thrombin injection is a viable alternative to traditional interventional radiology guided thrombin injection for management of pseudoaneurysms.

**Result(s):**

In clinic follow-up three weeks after thrombin injection, our patient continued to do clinically well, without abdominal pain or symptoms. Repeat CT angiogram demonstrated the GDA pseudoaneurysm had remained thrombosed, indicating successful endoscopic obliteration. He had no further signs of gastrointestinal bleeding.

**Conclusion(s):**

EUS guided thrombin injection is a novel approach that has been presented as an option in the management of bleeding gastroduodenal pseudoaneurysms, especially when radiology guided or surgical options are higher risk or have previously failed. The paucity of available data highlights the need for ongoing clinical studies comparing the different therapeutic strategies that can effectively and safely manage these rare but life-threatening vascular entities.

**Please acknowledge all funding agencies by checking the applicable boxes below:**

None

**Disclosure of Interest:**

None Declared

